# Health

**Published:** 1882-01

**Authors:** 


### HEALTH

A daily action of the bowels is essential to good health under all circumstances; the want of it engenders the most painful and fatal diseases. Nature prompts this action with great regularity, most generally after breakfast. Hurry or excitement will dispel that prompting, and the result is, nature is baffled. Her regular routine is interfered with, and harm is done. This is a thing which most persons do not hesitate to postpone, and in the case of riding to town, a delay of one or two hours is involved. This never can occur with impunity, in any single instance, to any person living. This very little thing—postponing nature's daily bowel actions—failing to have them with regularity—is the cause of all cases of piles and anal fistulas, to say nothing of various other forms of disease: fever, dyspepsia, headache, and the whole family of neuralgias. A man had better lose a dinner, better sacrifice the earnings of a day, than repress the call of nature; for it will inevitably lead to constipation, the attendant and aggravator of almost every disease. To arrange this thing safely, breakfast should be had at such an early time as will allow of a full half hour's leisure between the close of the meal and the time of leaving for the cars.

